# New Transfection Agents Based on Liposomes Containing Biosurfactant MEL-A

**DOI:** 10.3390/pharmaceutics5030411

**Published:** 2013-08-16

**Authors:** Mamoru Nakanishi, Yoshikazu Inoh, Tadahide Furuno

**Affiliations:** School of Pharmacy, Aichi Gakuin University, 1-100 Kusumoto-cho, Chikusa-ku, Nagoya 464-8650, Japan; E-Mails: inoh@dpc.agu.ac.jp (Y.I.); furuno@dpc.agu.ac.jp (T.F.)

**Keywords:** biosurfactant, cationic liposome, membrane fusion, siRNA, CLSM

## Abstract

Nano vectors are useful tools to deliver foreign DNAs, oligonucleotides, and small interfering double-stranded RNAs (siRNAs) into mammalian cells with gene transfection and gene regulation. In such experiments we have found the liposomes with a biosurfacant mannosylerythriol lipid (MEL-A) are useful because of their high transfer efficiency, and their unique mechanism to transfer genes to target cells with the lowest toxicity. In the present review we will describe our current work, which may contribute to the great advance of gene transfer to target cells and gene regulations. For more than two decades, the liposome technologies have changed dramatically and various methods have been proposed in the fields of biochemistry, cell biology, biotechnology, and so on. In addition, they were towards to pharmaceutics and clinical applications. The liposome technologies were expected to use gene therapy, however, they have not reached a requested goal as of yet. In the present paper we would like to present an approach using a biosurfactant, MEL-A, which is a surface-active compound produced by microorganisms growing on water-insoluble substrates and increases efficiency in gene transfection. The present work shows new transfection agents based on liposomes containing biosurfactant MEL-A.

## 1. Introduction

Gene transfection and gene regulation refers to the transmission of DNAs or small (21–23 nucleic acid) interfering double-stranded RNAs (siRNAs) to regulate specific gene expression in the target cells or organs and are very important in biosciences, pharmaceutics, and clinical applications. Over the past two decades, various non-viral vectors have been examined to avoid “Tragic setback” with virus vectors, such as adenoviruses and retroviruses [1]. However, the success of gene transfection and gene regulation by non-viral vectors did not proceed well because of their low transfection efficiency [2,3,4,5,6,7,8].

**Figure 1:**
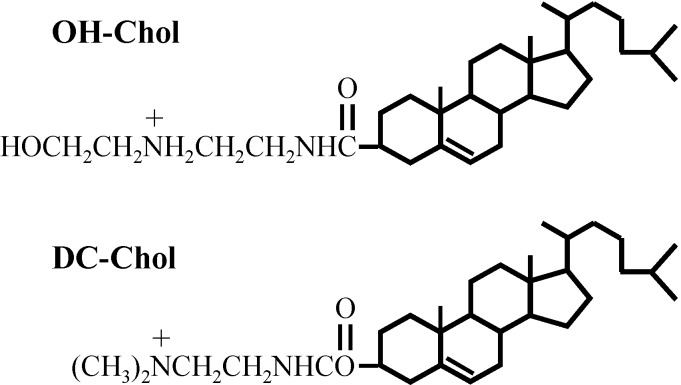
Structures of cholesteryl-3β-carboxyamidoethylene-*N*-hydroxyethylamine, OH-Chol and commercially available cationic cholesterol DC-Chol (lower).

Ten years ago, we reported a short review article for the non-viral liposome vectors made of cationic cholesterol derivatives [9]. Cationic cholesterol derivatives are composed of three distinct parts, a cholesteryl skeleton, a spacer arm between cationic amino residue and cholesterol, and a cationic amino terminal group. The derivatives with different combinations of these three parts were also reported and some of them have rather high transfection efficiency [4,5,6,7,8,9]. There, we found that the liposomes made of a cholesterol derivative with a hydroxyethyl amino head group are much more effective than those of the liposomes with the conventional cationic cholesterol derivative of alkylamino head groups as shown in Figure 1. It seemed that the liposome vectors must preserve the balance of molecular interaction between the hydrophilicity and hydrophobicity to transfer genes to the target cells [5]. Therefore, we studied the molecular mechanism of gene transfection with molecular imaging and that we found several accessory molecules with the liposomes were able to promote the transfer of DNAs into the target cells [9].

A biosurfactant, mannosylerythriol lipid A (MEL-A), is a major component of MELs produced by yeast strain *Candida antarctica* T-34 [10,11]. Biosurfactants have received great attention due to their unique properties such as low toxicity, biodegradability, biological activities, and self-assembling properties [12,13,14]. We found that MEL-A dramatically increased the efficiency of gene transfection, in 2001 [15], and clarified why the liposomes with MEL-A were able to increase the efficiency of the gene transfection [16]. The great transfection efficiency was due to the membrane fusion between liposomes and the target cells similar to some RNA virus vectors [16,17,18,19]. That is, the liposomes with MEL-A introduce foreign genes into the target cells by two different pathways; one is a pathway with the endocytosis of the vector/gene complexes into the target cells and another is a pathway of the membrane fusion, rapidly and safely.

In addition, RNA interference (RNAi) is mediated by siRNAs that originate from longer double-stranded DNAs (shRNAs) of endogenous or exogenous origin after cleavage by Dicer [20]. These siRNA molecules are then incorporated into a siRNA-induced silencing complex (RISC) where the shRNAs are cleaved to release single-stranded siRNAs [21,22,23]. RNAi is a promising tool for gene therapy against various diseases because siRNA has high targeting specificity for selective proteins without side effects [24,25,26,27]. Furthermore, siRNA is more stable and works at a lower concentration, for a longer time, than single-stranded oligonucleotides like antisense DNAs [28]. Despite these promising properties of siRNA therapeutics, several obstacles remain to be overcome. One of them is the poor cellular uptake of siRNA and accumulation of exogenous siRNA in endosomes [29]. Thus, a more efficient and safer system for siRNAs and shRNAs is needed for these molecules to make a significant clinical impact [30,31,32,33,34,35].

## 2. Liposome Vectors with Biosurfactants MEL-A

**Figure 2:**
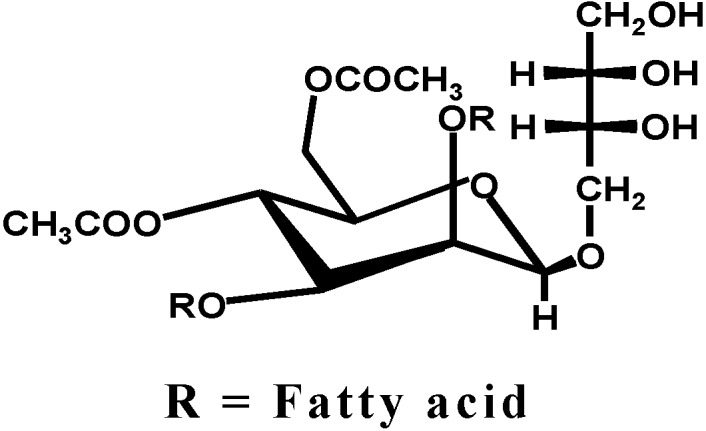
A structure of biosurfactant MEL-A which consists of 4-*O*-[β-d-mannopyranosyl] *meso*-erythritol], esterified two fatty acids and two acetic acids.

Non-viral liposome vectors were made of the complex of DOPE (dioleoyl phosphatidyl ethanolamine) and a cationic cholesterol derivative, cholesteryl-3β-carboxyamidoethylene-*N*-hydroxy ethylamine (OH-Chol) or commercially available cationic cholesterol (DC-Chol) to compare the gene transfection efficiency as also shown in Figure 1 [5,9]. A structure of the biosurfactant MEL-A, consisting of 4-*O*-(di-*O*-acetyl-di-*O*-alkanoyl-β-d-mannopyranosyl)-erythritol, esterified two fatty acids and two acetic acids and is shown in Figure 2.

**Figure 3:**
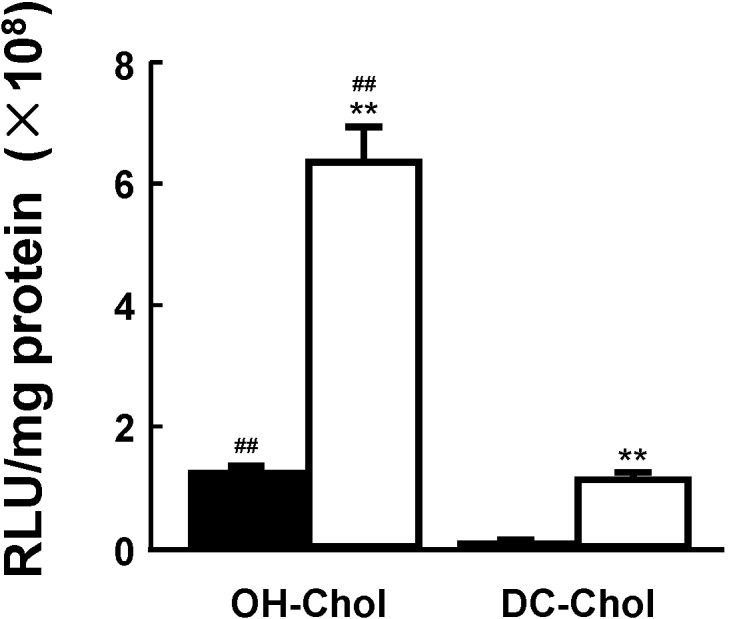
Effects of the liposome vectors with MEL-A on the transfection efficiency in target cells. NIH-3T3 cells were transfected with luciferase gene (pGL3) using two different cationic cholesterol derivatives, OH-Chol and DC-Chol without MEL-A (black bars) and with MEL-A (white bars). Transfection efficiency was measured by luciferase assay and values were normalized to the amount of total protein. Values are mean ± S.E. (*n* = 3). ** *p* < 0.01 compared with cationic liposomes without MEL-A; ^##^ *p* < 0.01 compared with DC-Chol by Students *t* test, respectively.The details were described in our previous paper [16].

MEL-A-containing liposome vectors with a cholesterol derivative OH-Chol or DC-Chol were complexed with plasmid DNAs for luciferase (pGL3) or siRNAs targeting luciferase (siGL3) (sense: 5'-CUUACGCUGAGUACUUCGATT-3' and antisense 5'-UCGAAGUACUCGCGUAAGTT-3'). Effects of transfection (not transfecting) activity were measured by luciferase activity or the silencing effects of target protein to the target cells [22,23]. The MEL-A greatly increased transfection activities in the target cells using both liposome vectors composed of OH-Chol and DC-Chol in comparison with the vectors without MEL-A as shown in Figure 3. In addition, the liposome vectors of OH-Chol had greater transfection efficiency than those of the DC-Chol.

In the experiments of siRNA silencing effects, we measured the suppression of Bcl-2 expression in B16/BL6 melanoma cells after delivering siRNA targeting Bcl-2 by MEL-A-containing cationic liposomes. To determine the most effective method of siRNA transfection, we measured cytotoxicity and suppression of luciferase expression in luciferase-expressing B16 cells (B16-luc cells) exposed to siGL3 at a fixed concentration with various ratios of MEL-A-containing cationic liposomes. The details were described in the previous paper [36]. As described above, such kinds of characteristics existed both in the case of plasmid DNAs and siRNAs (shRNAs) with liposome vectors containing biosurfactant MEL-As [36].

To investigate the cellular uptake mechanism of siRNAs, we studied the subcellular localization of fluorescein-labeled siRNAs after transfection with confocal laser scanning fluorescence microscopy (CLSM). The results revealed that MEL-A-containing cationic liposomes were able to deliver siRNA into the cytoplasm of the target cells very rapidly, and that a short incubation time of 10–30 min was enough to reach the value of the maximum silencing effect, though Lipofectamine™ RNAiMax (Invitrogen, Carlsbad CA, USA) needed much more time and the silencing effect increased more slowly [36].

## 3. Mechanism to Transfer the Liposome/Vector Complexes to Target Cells

CLSM gave us useful information on how the liposome vectors with MEL-A are very effective to transfer plasmid DNAs and siRNAs into the target cells. Another microscopic method, atomic force microscopy (AFM) is also very useful to understand the molecular mechanism of the gene transfection and the structure of the complex of non-viral vectors and genes. As we described the experimental results by AFM in our previous review article and several papers [9,37,38], we do not describe them in the present paper.

**Figure 4:**
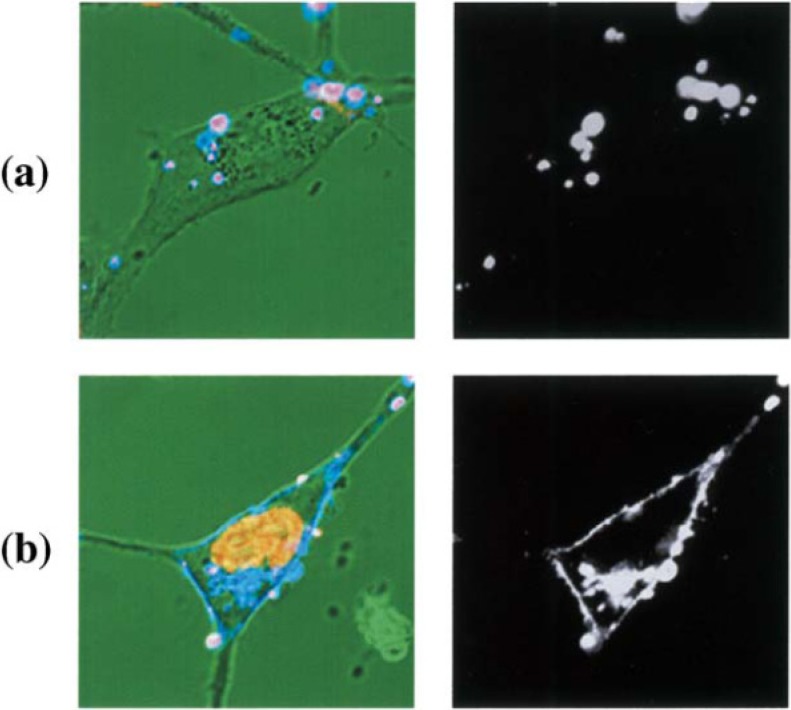
CLSM images of NIH3T3 cells incubated with fluorescence labeled liposome-DNA complex. NIH3T3 cells were incubated with FITC-conjugated oligonucleotides complexed with rhodamine-labeled liposomes composed of OH-Chol without MEL-A (**a**) and with MEL-A (**b**) for 1 h. Fluorescence images of liposome (**right**) and merged images on a transmittance image with pseudo-color (yellow for DNA, blue for liposome, and pink for liposome-DNA-complex) (**left**) are shown.

In the present paper we will describe the interesting and unique results of the gene transfection with non-viral vectors with MEL-A through the fluorescence energy transfer and the fluorescence *in vitro* cell images. Figure 4 shows CLSM images of NIH3T3 cells incubated with fluorescence labeled liposome-DNA complex, using OH-Chol as a cholesterol derivative. In the case of liposomes with MEL-A, fluorescence labeled liposomes with MEL-A/antisense DNA complexes were temporarily on the plasma membrane of the target cells, and antisense oligonucleotides were transferred into the nucleus. The distribution of bright fluorescence was observed along the plasma membranes. This suggested that membrane fusion occurred between liposomes and the plasma membrane. To the contrary, the fluorescence intensity change was neither observed in the nucleus nor on the plasma membranes at 1 h incubation with the cationic liposomes without MEL-A [16].

**Figure 5:**
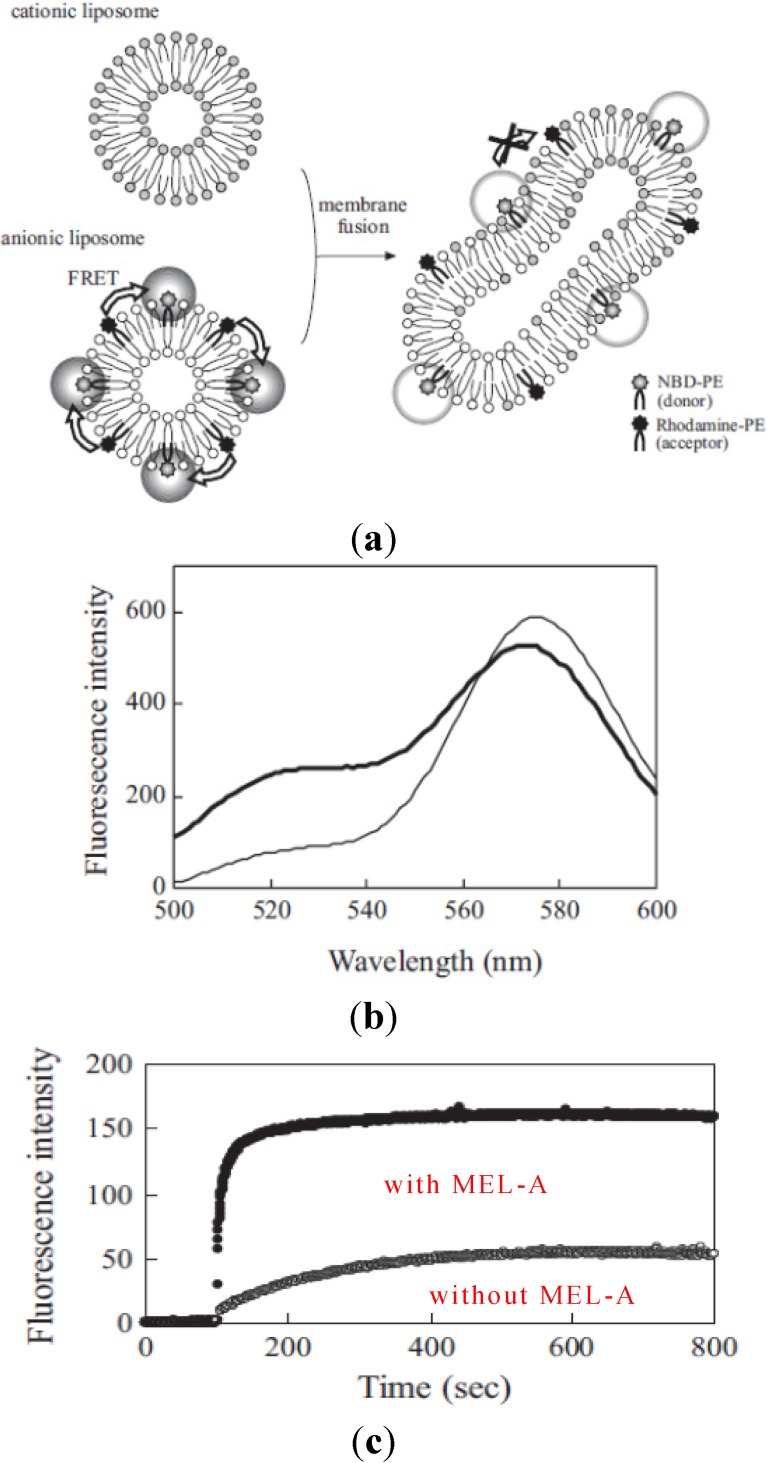
The decrease in FRET efficiency by membrane fusion from NBD-PE to rhodamine-PE. (**a**) The schematic representation of decrease in FRET efficiency from NBD-PE to rhodamine-PE by membrane fusion; (**b**) Fluorescent spectra of NBD and rhodamone in anionic liposomes before (broken line) and at 10 min after the addition of cationic liposomes containing MEL-A (solid line); (**c**) The time course of membrane fusion between cationic and anionic liposomes by FRET assay. The cationic liposomes with MEL-A intensity of NBD according to the decrease in FRET efficiency by member fusion were monitored.

To understand more precisely how membrane fusion occurred between liposome membranes and the target cell membranes, we used two kinds of model membranes to study the effects of biosurfactants on the fluorescence energy transfer. As shown in Figure 5, we studied the effects of MEL-A on the membrane fusion between cationic liposomes and anionic model liposomes by fluorescence spectroscopy. The procedure is shown in Figure 5, schematically [16]. The membrane fusion was measured with the decrease in FRET efficiency between two fluorophores, NBD-PE (donor) and rhodamine-PE (acceptor) in anionic liposomes according to the increase of their distance by mixing with non-fluorescent lipids in cationic liposomes. Therefore, when the membrane fusion occurred between anionic liposomes and cationic liposomes, NBD fluorescence intensity increased at 525 nm and rhodamine fluorescence at 575 nm decreased as shown in Figure 5. Figure 5c shows the time course of fluorescence intensity changes of NBD at 525 nm in anionic liposomes after the addition of cationic liposomes with or without MEL-A. The plateau level of fluorescence intensity after the addition of liposomes with MEL-A (after 10–15 min) increased much more than that in the case without biosurfactants. These results were well consistent with our previous findings that MEL-A-containing cationic liposome membranes were able to facilitate the fusion between the plasma membrane of the target cells and cationic liposomes [16].

## 4. Membrane Fusion Works for siRNA Transfection

**Figure 6:**
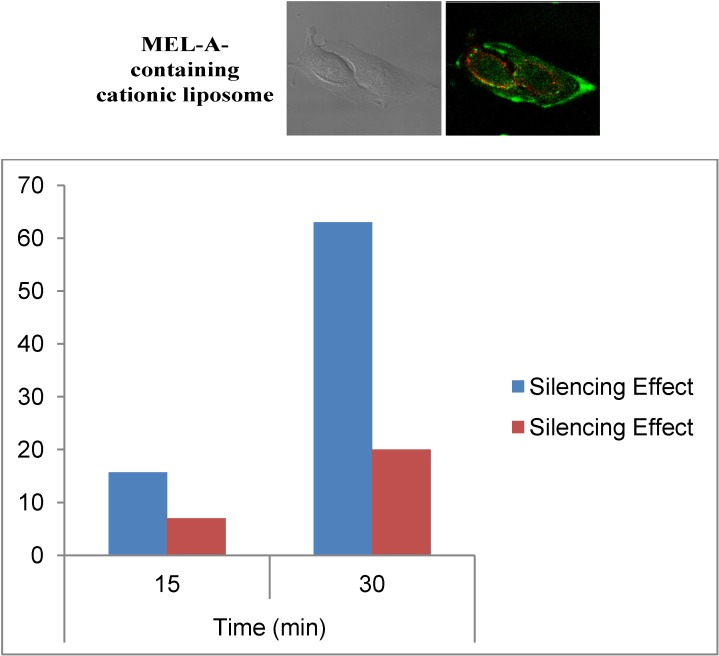
(**Upper**) Intracellular localization of FAM-labeled siRNA with MEL-A-containing cationic liposomes. Lysotracker Red stained B16/BL6 cells were observed 0.5 h after incubation with liposome/siRNA. **Left** is DIC and **Right** is a CLSM image of FAM-labeled siRNA; (**Lower**) Time-depended silence effects for the experiments with MEL-A-containing cationic liposomes (blue) and Lipofectamine™ RNAiMax (red).

Figure 6 shows a typical example of CLSM images of B16/BL6 cells treated with MEL-A-containing liposome/siRNAs complexes described above. Here, MEL-A-containing liposome vectors with a cholesterol derivative OH-Chol were complexed with siRNAs. Endosomes/lysosomes were stained with LysoTracker Red (red) and siRNAs were stained by fluorescein-conjugated siRNA (green). The details of the experimental procedure were reported in a previous paper [36]. As shown in Figure 6, most of the fluorescein-conjugated siRNA fluorescence (green) was separated from endosomes/lysosomes fluorescence (red) when B16/BL6 cells were treated with the MEL-A-containing liposome/siRNAs complexes [36]. The results indicated that MEL-A-containing cationic liposomes were able to deliver siRNAs rapidly into the target cells within 10–30 min of incubation, time shown in Figure 6. It was quite different from the siRNAs that were transfected with Lipofectamine™ RNAiMax. Here, siRNAs were transferred much slower into the target cells with Lipofectamine™ RNAiMax, although, after 1~2 h, both methods reached almost similar levels of silencing effects. This indicated that the cellular uptake mechanism of siRNAs mediated by MEL-A-containing cationic liposomes was quite different from those mediated with Lipofectamine™ RNAiMax, where the endocytotic pathway was mainly involved in gene transfer to the target cells.

## 5. Intracellular Rapid Distribution of Components of Liposome/siRNA Complex

Finally, we investigate the mechanism of the rapid delivery of siRNAs to the target cells. We studied the individual processes in delivery of siRNAs into the target cells with the liposomes/siRNAs complexes of MEL-A. The typical fluorescence images in siRNAs complexes were transferred into the target cells by the MEL-A-containing cationic liposomes shown in Figure 6. At 10 min after the addition of the liposome-DNA complexes, they attached to the plasma membrane of target NIH-3T3 cells. A small amount of MEL-A was found to start to distribute to the intracellular membranes. Rhodamine-conjugated PE, which was a content of the vector, was diffused from the contact site to the plasma membrane of target cells, indicating that the fusion between the liposome and the plasma membrane occurred. Figure 5c shows representative time-courses of fluorescence intensity changes of NBD-conjugated MEL-A in intracellular membranes, rhodamine-conjugated PE in the plasma membrane, and Cy5-conjugated oligonucleotide in the nucleus. The MEL-A was accumulated in the intracellular membranes continuously after the addition of the complexes, while the fusion of the vectors with the plasma membrane proceeded. After a short period of time, the oligonucleotide DNA was suddenly released from the complexes by the membrane fusion and it was delivered into the nucleus. A schematic model of gene transfer with MEL-A-containing liposome vectors, based on the present experiment, is shown in Figure 5. The membrane fusion between the plasma membranes and the liposome vectors plays a role in gene transfection in the case of liposome vector with MEL-A.

## 6. Conclusions

Biosurfactants are extracellular amphiphilic compounds produced by various kinds of microorganisms and have attracted considerable interest because of their biodegradability, mild productive conditions, and variety of other functions. The numerous advantages of biosurfactants have promoted applications in food, cosmetic, and pharmaceutical industries, and environmental protection and energy-saving technology [10,11,12,13,14]. It is also considered that biosurfactants play important roles as immune-regulators and immune-modulators in adhesion and desorption in cellular systems. In addition to these interesting properties of biosurfactants, we showed that the biosurfactant MEL-A-containing liposome vectors remarkably promoted the efficiency of gene transfection into mammalian cultured cells [15,16].

Further, we showed in the present review that the MEL-A-containing liposome vectors transfected foreign genes into the nucleus of target cells with a shorter incubation time (10~30 m) via membrane fusion. The liposome vector has a new pathway of the membrane fusion to transfer genes to the target cells with high rapidity and efficiency, which seems a great technique to avoid the “Tragic setback” with virus vectors [1].
